# Exploratory study on the potential benefits of prophylactic levetiracetam after acute spontaneous intracerebral hemorrhage

**DOI:** 10.3389/fneur.2025.1580539

**Published:** 2025-08-20

**Authors:** Marc A. Babi, Irina Lishen, Barbara Pierre Louis, Katelyn Woodbury, Anthony Kennedy, Fouad Abuzeid, Amre Nouh

**Affiliations:** ^1^Department of Neurology, Cleveland Clinic Florida/Martin Health, Port Saint Lucie, FL, United States; ^2^Department of Neurology, Cleveland Clinic Florida, Weston, FL, United States; ^3^Case Western Reserve University of Cleveland Clinic Lerner College of Medicine, Cleveland, OH, United States; ^4^Department of Pharmacy, Cleveland Clinic Florida/Martin Health, Port Saint Lucie, FL, United States

**Keywords:** neurocritical care, seizures, critical care, intracranial hemorrhage, intracerebral hemorrhage, intensive care unit, levetiracetam

## Abstract

**Importance:**

Current guidelines recommend against the routine use of seizure prophylaxis in acute spontaneous intracerebral hemorrhage (sICH).

**Objective:**

The goal of this study is to evaluate if the use of prophylactic levetiracetam resulted in reduced incidence of seizure, morbidity, and length of stay, compared to patients who did not receive prophylactic levetiracetam.

**Design:**

This retrospective chart review includes patients admitted with ICH at Cleveland Clinic Florida Martin Health from January 2019 to October 2022.

**Main outcome and measures:**

The primary outcome was the incidence of seizure during the first 7 days of admission. Secondary outcomes include intensive care unit and hospital length of stay, measured in days.

**Results:**

A total of 160 patients were included in this study, 93 in the levetiracetam group. The primary outcome of clinical seizure incidence within 7 days of ICH diagnosis was observed in five patients, all of whom were in the prophylactic levetiracetam group, though this did not reach statistical significance (5% vs. 0%; *p* = 0.075). In adjusted analysis, levetiracetam showed a non-significant protective trend (OR 0.71, 95% CI 0.13–3.79). The median length of hospital stay and ICU stay were both longer in the prophylactic levetiracetam group (5 days vs. 3 days; *p* < 0.001 and 2 days vs. 1 day; *p* = 0.001, respectively). However, in adjusted analyses, these differences were not statistically significant.

**Conclusion and relevance:**

The routine use of seizure prophylaxis with levetiracetam did not result in a significant reduction in early seizure incidence, and in unadjusted analyses, the prophylaxis group had longer ICU and hospital stays. However, these differences were not significant after adjustment for key clinical confounders. Randomized controlled trials need to be conducted to determine whether seizure prophylaxis with levetiracetam contributes to worse outcomes.

## 1 Introduction

Spontaneous intracerebral hemorrhage (ICH) is associated with morbidity and mortality as high as 30 to 50% (1–3). It is estimated that ICH accounts for 10 to 15% of strokes per year (1, 4). One of the common early complications of ICH is acute symptomatic seizures, occurring within 7 days of stroke onset, as defined by the International League Against Epilepsy (1, 4). The occurrence of seizures has been associated most frequently with lobar hemorrhage (4). Studies have shown that acute symptomatic seizures are associated with worse functional outcome and increased mortality risk in patients with ICH due to an increase in metabolic demand, change in hematoma volume, and midline shift (5, 6). However, some other studies suggest that the association between symptomatic seizures and outcomes remains incompletely established (1, 7, 8). Reconciling these apparently conflicting results requires careful analysis of patient selection, timing, and methodology, and underscores the need for additional research. Our understanding of risk factors for developing seizures and the role of antiseizure medications in preventing poststroke seizures is limited to a few retrospective studies. Current information regarding the efficacy of seizure prophylaxis post-ICH has been controversial. The American Heart Association/American Stroke Association (AHA/ASA) guideline states that in patients with spontaneous ICH without evidence of seizures, prophylactic antiseizure medication is not beneficial to improve functional outcomes, long-term seizure control, or mortality (9). Despite these guidelines, the use of antiseizure medications in ICH patients varies between institutions and practitioners. One survey including 199 physicians reported that 32% of neurologists, 11% of neurosurgeons, and 57% of intensivists routinely use seizure prophylaxis in patients with spontaneous ICH, with levetiracetam as the most common drug choice (10). A recent double-blind, randomized, placebo-controlled phase 3 trial at three stroke units in France found that levetiracetam might be effective in preventing acute seizures in ICH (11). Given its favorable safety profile, low protein binding, and metabolism that is largely independent of liver function, levetiracetam has emerged as a common prophylactic choice in this population. However, evidence supporting or refuting its use in preventing seizures after acute spontaneous ICH is still evolving. At our institution, prophylactic levetiracetam is often prescribed for patients considered higher risk (e.g., with lobar ICH, larger hematomas, or midline shift). This local practice potentially introduces indication bias, because patients who are perceived to be at higher risk of seizures by treating clinicians may be more likely to receive prophylaxis.

To date, there is limited data on the impact of prophylactic antiseizure medications on clinical outcomes in patients with ICH. The goal of this exploratory study is to examine adult patients with a primary diagnosis of spontaneous ICH and investigate if prophylactic levetiracetam leads to a reduction of seizure activity and a change in clinical outcomes, specifically reduced intensive care unit (ICU) and hospital length of stay (LOS), compared to no prophylaxis.

## 2 Materials and methods

### 2.1 Description of study design and participants

This was a multicenter, retrospective chart review of all adult patients with ICH admitted to the Cleveland Clinic Florida Martin Health System (CCMHS) from January 2019 to October 2022. This study was approved by our Institutional Review Board (IRB) Committee through WCG-IRB as an exempt study under 45 CFR § 46.104(d)(4), study protocol: 1267. No funding was obtained to conduct this study. The IRB committee determined that participants’ consent was not required or waived, due to the retrospective nature of this study.

Cleveland Clinic Florida Martin Health System (CCMHS) is a multicenter, academic network located in South Florida and is fully integrated as part of the Cleveland Clinic Healthcare System. This hospital consists of a 521-bed, not-for-profit system which includes three acute care sites: North, South, and Tradition campuses. Electronic medical records (Epic®) were reviewed to identify patients using the 10th version of the International Classification of Diseases (ICD) for non-traumatic ICH and then further divided into two groups: patients who received prophylactic doses of levetiracetam and patients who did not.

#### 2.1.1 Inclusion criteria

1. Age ≥18 years.
2. Admitted to CCMHS with an ICH diagnosis.
3. Hospitalized for ≥48 h.

#### 2.1.2 Exclusion criteria

1. Hemorrhage resulting from encephalitis, trauma, ruptured aneurysm or vascular malformation, brain tumor, subarachnoid hemorrhage, or hemorrhagic transformation after thrombolytic administration;
2. Invasive neurosurgical procedure;
3. Transfer to another facility or left against medical advice;
4. History of seizures or antiseizure medication use in the 4 weeks prior to the onset of ICH;
5. Antipsychotic medication use prior to admission;
6. Transition to hospice-care status or withdrawal of artificial support within 7 days of admission;
7. Pregnancy or age <18 years.

A seizure was defined as (1) a clinical event consistent with epileptic activity (e.g., tonic–clonic convulsion or focal onset with impaired awareness) and/or (2) electroencephalography (EEG) findings of ictal or interictal epileptiform discharges at a frequency >2.5 Hz.

Mean ICH volume, GCS, ICH score, and other characteristics among both groups are reported in Table 1.

**Table 1:** Characteristics overall and demographic of total participants.

Data	Total *n* = 160	No	Yes	*p*-value
		Prophylactic levetiracetam *n* = 67	Prophylactic levetiracetam *n* = 93	
Age (years)^a^	75 [64–84]	76 [67–86]	74 [62–83]	0.096
Sex (female)^b^	81 (51)	33 (49)	48 (52)	0.768
Race^b^				0.697
Black	19 (12)	6 (9)	13 (14)	
Hispanic	15 (9)	6 (9)	9 (10)	
Unknown	4 (3)	1 (2)	3 (3)	
White	122 (76)	54 (81)	68 (73)	
ICH location, non-lobar^b^	95 (59)	53 (79)	42 (45)	<0.001
Hydrocephalus^b^	2 (1)	2 (3)	0 (0)	0.094
Intraventricular hemorrhage^b^	31 (19)	13 (19)	18 (19)	0.994
Midline shift^b^	22 (14)	4 (6)	18 (19)	0.015
Mean ICH Volume (mL)		17.3 (6.8)	15.6 (7.2)	<0.001
Admission GCS^a^	15 [14–15]	15 [15–15]	15 [13–15]	<0.001
Admission NIH stroke scale^a^	2 [0–6]	1 [0–3]	3 [0–9]	<0.001
Other AEDs	5 (3)	0 (0)	5 (5)	0.054
ICH Score^a^	1 [0–1]	1 [0–1]	1 [0–1]	0.876
EEG monitoring^b^	27 (17)	4 (6)	23 (25)	0.002
Renal function CrCl^b^				0.115
<15	5 (3)	3 (4)	2 (2)	
15–29	7 (4)	4 (6)	3 (3)	
30–44	17 (11)	8 (12)	9 (10)	
45–59	32 (20)	12 (18)	20 (22)	
60–89	59 (37)	30 (45)	29 (31)	
≥90	40 (25)	10 (15)	30 (32)	

Presented as ^a^median [IQR] ^b^*n* (%). CrCl, Creatinine clearance; GSC, Glasgow Coma Scale; NIH, National Institutes of Health; AED, Antiepileptic drugs; EEG, Electroencephalogram.

### 2.2 Outcomes and analysis

The primary outcome of this study was the incidence of seizure activity during the first 7 days of admission. Secondary outcomes included intensive care unit and hospital length of stay, measured in days. Delayed loading of levetiracetam (i.e., a loading dose given beyond 1 h of the intended time) was noted and further reviewed to determine potential reasons, which included administrative delays, intravenous access issues, or uncertainty regarding the risk of seizures on initial presentation.

Categorical variables are presented as frequencies with proportions. Group differences were analyzed by chi-squared and Fisher’s exact test depending on expected cell frequencies. Continuous variables are presented as means with standard deviations or medians with interquartile ranges (IQR) depending upon the normality of the data distribution. Normality was determined by Shapiro–Wilk’s test. Group differences were analyzed by Student’s t-test or non-parametric equivalent as appropriate. Significance was defined as *p* < 0.05. Analysis was performed using STATA version 16.1.

## 3 Results

**Figure 1:**
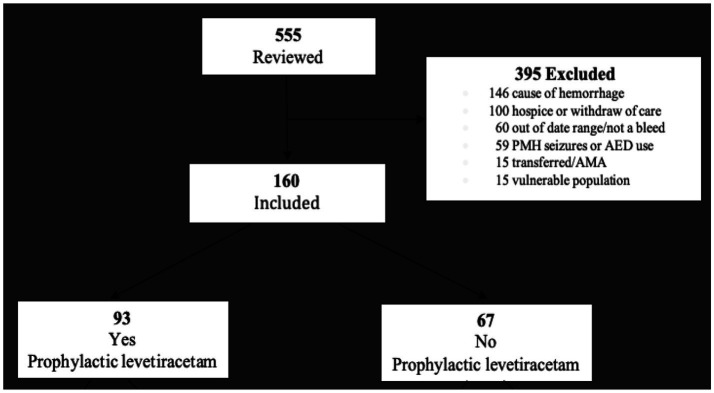
Cohort selection flow diagram. All patients in the initial population were adults (≥18 years) presenting to our facility (Cleveland Clinic Florida Martin Health) of an integrated-academic medical center between January 2019 and October 2022.

A total of 555 charts were reviewed, and 160 met the inclusion criteria. The most common reasons for exclusion were etiology of hemorrhage (i.e., non-spontaneous ICH, trauma, and post-operative hemorrhage). Other reasons for exclusion are listed in Figure 1. Of the 160 included, 93 patients received prophylactic levetiracetam and 67 did not.

The study population was primarily female (51%), white (76%), with a median age of 75 years. Baseline characteristics were similar between groups with a few exceptions (Table 1). Patients who were not receiving prophylactic levetiracetam had a higher proportion of non-lobar ICH location (79% vs. 45%; *p* < 0.001) and a higher median admission GCS (15 [15–15] vs. 15 [13–15]; *p* < 0.001) compared to those receiving prophylactic levetiracetam. Those receiving levetiracetam had a higher median NIH stroke scale (3 [0–9] vs. 1 [0–3]).

Most patients receiving prophylactic levetiracetam received their first dose on hospital day zero (87%) in the emergency department (73%). The most administered dose at continuation was 500 mg (twice daily) (81%), and the median duration of therapy was 4 days. Three patients received a late levetiracetam loading dose, defined as being late if it was given after 1 h of the scheduled time, and the number of missed doses at continuation was four, two patients missing one dose and one patient missing three doses. Levetiracetam was discontinued due to behavioral disturbances in four patients. Of the five patients who had a seizure, three patients’ levetiracetam dose was increased. A total of 38% of patients were discharged on levetiracetam. For more details regarding prophylactic levetiracetam characteristics, refer to Table 2.

**Table 2:** Characteristics of medication administration Prophylactic levetiracetam characteristics.

Data	Total *n* = 93
Hospital day of first levetiracetam dose^a^	
0	81 (87)
1	8 (9)
2	4 (4)
Location of first levetiracetam dose^a^	
ED	68 (73)
ICU	24 (26)
PCU	1 (1)
Median total daily dose of levetiracetam, *n* = 91^b^	1,000 [1000–1,000]
Total daily dose of levetiracetam, *n* = 91^a^	
250	1 (1)
500	5 (5)
1,000	75 (81)
1,500	5 (5)
2,000	4 (4)
3,000	1 (1)
Duration of levetiracetam therapy, *n* = 91^b^	4 [3–7]
Number of late loading doses of levetiracetam^a^	3 (3)
Number of missed continuation doses of levetiracetam^a^	
1	2 (2)
3	1 (1)
Discontinued due to behavior disturbance^a^	4 (4)
Increased dose in those with seizures observed^a^	3/5 (60)
Levetiracetam prescribed at discharge^a^	35 (38)

Presented as ^a^median [IQR] ^b^*n* (%).

The primary outcome of seizure incidence within 7 days of ICH diagnosis and hospitalization was observed in five patients, all of whom were in the prophylactic levetiracetam group (Table 3). There was no statistically significant difference in the primary outcome of seizure incidence between those receiving prophylactic levetiracetam compared to those who did not (5% vs. 0%; *p* = 0.075). Of those who experienced a seizure, four patients experienced one seizure and one patient experienced three seizures. The median length of hospital stay and ICU stay were significantly longer in the prophylactic levetiracetam group (5 days vs. 3 days; *p* < 0.001 and 2 days vs. 1 day; *p* = 0.001, respectively). However, after we performed multivariable negative-binomial regression models (chosen after testing for over-dispersion) with ICU LOS and hospital LOS as dependent variables, the same prespecified covariates used in our seizure model—lobar location, midline shift, and admission NIHSS (per 5-point increase)—were entered along with age and admission GCS; the difference in hospital and ICU LOS were no longer significant after adjustment for key clinical confounders:

- ICU LOS – Incidence Rate Ratio (IRR) 1.12, 95% CI 0.92–1.36, *p* = 0.26.
- Hospital LOS – IRR 1.07, 95% CI 0.90–1.27, *p* = 0.44.

**Table 3:** Outcomes overall and discharge disposition of included patients.

Data	Total *n* = 160	No	Yes	*p*-value
		Prophylactic levetiracetam *n* = 67	Prophylactic levetiracetam *n* = 93	
Seizure incidence within 7 days of ICH diagnosis and hospitalization^a^	5 (3)	0 (0)	5 (5)	0.075
Number of observed or recorded seizures^a^				0.140
1	4 (3)	0 (0)	4 (4)	
3	1 (1)	0 (0)	1 (1)	
ICU length of stay^b^	2 [1–3]	1 [1–2]	2 [1–2]	0.001
Hospital length of stay^b^	4 [3–7]	3 [2–5]	5 [3–8]	<0.001
Discharge disposition^a^				0.332
Acute care	1 (1)	0 (0)	1 (1)	
Expired	3 (2)	2 (3)	1 (1)	
Home	64 (40)	31 (46)	33 (35)	
Hospice	4 (3)	2 (2)	2 (2)	
Rehab	67 (42)	22 (33)	45 (48)	
SNF	21 (13)	10 (15)	11 (12)	

Presented as ^a^median [IQR] ^b^*n* (%). SNF, skilled nursing facility.

Therefore, after adjustment, the apparent prolongation of LOS in the prophylaxis group was no longer statistically significant, confirming that the unadjusted differences were likely driven by baseline severity and location. Discharge disposition was not different between groups. The majority of patients went to rehab (42%) or home (40%).

Furthermore, after performing a multivariable logistic-regression analysis with the four prespecified covariates (prophylactic levetiracetam, lobar location, midline shift, and admission NIHSS—modeled per 5-point increase). With only five events, exact (conditional) logistic regression and Firth bias-reduced methods were both tested; both produced nearly identical point estimates and wide CIs. Levetiracetam shows a non-significant trend toward protection once confounding by lobar location and mass effect is accounted for. This data are summarized below:

Results (*n* = 160):

- LEV prophylaxis – adjusted OR 0.71 (95% CI 0.13–3.79, *p* = 0.69).
- Lobar ICH – adjusted OR 4.02 (1.09–14.8), *p* = 0.036.
- Midline shift – adjusted OR 3.54 (0.97–12.9), *p* = 0.055.
- NIHSS (per 5 points) – adjusted OR 1.32 (0.89–1.96), *p* = 0.17.

## 4 Discussion

In this exploratory study, on unadjusted analysis, prophylactic levetiracetam was not associated with fewer clinically evident seizures; however, in an adjusted model that accounts for lobar location, midline shift, and stroke severity, levetiracetam showed a non-significant protective trend (adjusted OR 0.71). Patients receiving prophylaxis demonstrated a significantly prolonged hospital and ICU stay, possibly reflecting higher baseline severity—such as lobar ICH, midline shift, or higher NIH stroke scale—and potential indication bias in prescribing practices. A variety of factors could explain this longer length of stay in the levetiracetam group, including (1) higher incidence of lobar ICH, (2) occurrence of seizures, (3) midline shift, (4) higher admission NIH stroke scale, and (5) use of additional antiseizure medications. Each of these factors can heighten clinical complexity and highlight the confounding influence of disease severity on outcomes.

Our seizure definition required clinical correlation and/or EEG confirmation. However, the duration of continuous EEG monitoring was variable among patients, as 24-h continuous EEG was not performed for all ICH patients. Logistical challenges sometimes delayed connecting patients to continuous EEG monitoring after clinical seizures. As a result, subclinical seizures—an important contributor to morbidity in acute brain injury—may have gone undetected. This underestimation of seizure burden could alter the apparent impact of prophylaxis and underscores the importance of systematic EEG monitoring in future research.

Another possible explanation for the higher seizure incidence at 7 days is that clinicians preferentially started prophylaxis in “high-risk” patients who had lobar ICH, larger hematomas, or midline shift. This selection bias could have concentrated inherently on the higher seizure risk in the prophylaxis group. Alternatively, incomplete or delayed loading of levetiracetam in certain high-risk patients might have undermined its efficacy. Future prospective, randomized studies are needed to clarify whether levetiracetam truly prevents seizures in a general ICH population or merely reflects prescriber bias. Furthermore, different prescribing patterns across specialties—such as emergency medicine, neurosurgery, neurocritical care, and vascular neurology—may also influence the primary and secondary findings of this study.

Because prophylactic anticonvulsant initiation was not randomized and was left to the physician discretion, indication bias is likely. We note that the majority of patients received 500 mg BID, well below the 40–60 mg/kg loading and daily doses used in status epilepticus. However, the above dosage was used mainly prophylactically. We acknowledge that underdosing could blunt any prophylactic effect and may partially explain the neutral primary result. Patients with lobar ICH, larger hematomas, or other high-risk features may have been more likely to receive a higher prophylactic dose. Moreover, our definition of “seizure” focused on clinically evident events, thereby potentially missing purely electrographic or subclinical seizures that carry their own morbidity in acute brain injury.

Our findings align with the AHA/ASA guideline statements cautioning against routine seizure prophylaxis in ICH patients without seizures (9). Nevertheless, other studies show conflicting results: some suggest that prophylaxis may reduce seizure incidence (11), whereas others highlight that it may not improve—and may even worsen—functional outcomes (9). A study by Peter-Derex et al. demonstrated that in a center where cEEG is routinely used, low-dose levetiracetam (500 mg BID) appears safe and may reduce early subclinical seizures by 60 to 80%. The authors concluded that although there is no evidence that prophylaxis improves long-term disability or mortality, future trials must enroll larger, more diverse ICH populations and examine whether seizure prevention translates into sustained long-term improved neurological outcomes (12–16).

These inconsistencies across the literature likely reflect varying patient populations, differences in antiseizure regimens, and heterogeneity in EEG monitoring practices.

With the Florida population aging and the increased prevalence of anticoagulant use, the number of patients with ICH is expected to rise. Future high-quality randomized controlled trials that focus on lobar ICH will be critical in determining whether prophylactic levetiracetam truly affects outcomes.

## 5 Conclusion

In this exploratory study, prophylactic levetiracetam did not demonstrate a significant reduction in clinically apparent seizure incidence among patients with acute spontaneous ICH. While patients receiving prophylaxis had apparent longer ICU and hospital stay—possibly explained by more severe baseline disease or indication bias, after adjustment for other variables, this trend was not associated with a significant change in ICU or hospital length of stay. These results neither definitively support nor refute the role of levetiracetam in seizure prophylaxis for ICH. Future randomized, controlled trials with adequate sample sizes, routine continuous EEG monitoring, and multivariate statistical modeling are required to determine whether levetiracetam prophylaxis improves—or worsens—clinical outcomes in this population.

## Data Availability

The original contributions presented in the study are included in the article/supplementary material, further inquiries can be directed to the corresponding author.
